# Molecular mechanism of diabetic cardiomyopathy and modulation of microRNA function by synthetic oligonucleotides

**DOI:** 10.1186/s12933-018-0684-1

**Published:** 2018-03-22

**Authors:** Nilanjan Ghosh, Rajesh Katare

**Affiliations:** Department of Physiology-HeartOtago, University of Otago, 270, Great King Street, Dunedin, 9010 New Zealand

**Keywords:** Diabetic cardiomyopathy, MicroRNA, Modulation of microRNA, Delivery of therapeutic microRNA, Clinical application of microRNA

## Abstract

Diabetic cardiomyopathy (DCM) is a chronic complication in individuals with diabetes and is characterized by ventricular dilation and hypertrophy, diastolic dysfunction, decreased or preserved systolic function and reduced ejection fraction eventually resulting in heart failure. Despite being well characterized, the fundamental mechanisms leading to DCM are still elusive. Recent studies identified the involvement of small non-coding small RNA molecules such as microRNAs (miRs) playing a key role in the etiology of DCM. Therefore, miRs associated with DCM represents a new class of targets for the development of mechanistic therapeutics, which may yield marked benefits compared to other therapeutic approaches. Indeed, few miRs currently under active clinical investigation, with many expressing cautious optimism that miRs based therapies will succeed in the coming years. The major caution in using miRs based therapy is the need to improve the stability and specificity following systemic injection, which can be achieved through chemical and structural modification. In this review, we first discuss the established role of miRs in DCM and the advances in miRs based therapeutic strategies for the prevention/treatment of DCM. We next discuss the currently employed chemical modification of miR oligonucleotides and their utility in therapies specifically focusing on the DCM. Finally, we summarize the commonly used delivery system and approaches for assessment of miRNA modulation and potential off-target effects.

## Background

Recent research findings show that more than 70% of diabetic individuals will develop some form of heart disease during their life span [1]. Importantly, diabetes itself is an independent risk factor for cardiovascular disease [2–8]. Population-based studies have revealed that the risk of heart failure is augmented 2 to 3-fold by diabetes [9, 10]. The occurrence of diabetes considerably accelerates the development of heart failure in patients with myocardial infarction [11, 12], hypertension [13], or atrial fibrillation [14]. The impact of diabetes on the development of vascular disease have been established earlier but non-ischemic heart failure associated with diabetes termed as diabetic cardiomyopathy (DCM) has received fewer attention than coronary and cerebrovascular events. Identification of the disease modulators and its translation can help us to develop novel therapeutic strategies of this global burden. MicroRNAs (miRs) a novel class of non-coding RNAs have recently been evolved as a key role player in various cardiovascular diseases including DCM. Further, therapeutic modulation of miRs has gained some interest in diabetic heart disease research, but a vast area in this research line is still unexplored. In this review, we discuss the pathophysiology of DCM focusing on the pathological role of miRs. We next summarize the recent advances in the therapeutic modulation of miRs with a special focus on the available techniques to modulate miRs with the aim to provide a foundation for future research studies developing novel miRs based therapy to prevent/treat DCM.

### Diabetic cardiomyopathy (DCM)

According to National Institute of Health (NIH) and American Heart Association (AHA), cardiomyopathy is a collective term, which refers to the disease of myocardium that leads to chronic and progressive damage. Several factors can trigger the development of abnormal structural and functional alterations which altogether leads to the development of heart failure [15–17].

DCM was first discovered during an autopsy performed in a patient diagnosed with diabetic-glomerulo-sclerosis [18]. In spite of being discovered almost four decades ago, a considerable amount of information on its pathogenesis and clinical manifestation is still not clear. Diabetic heart exhibits enhanced fatty acid catabolism and reduced glucose metabolism leading to insufficient energy production. This provokes the excitation–contraction coupling in the diabetic heart increasing its susceptibility to ischemia–reperfusion injury [19]. Further, diabetes also induces microvascular rarefaction in the heart thereby diminishing the coronary perfusion, eventually resulting in contractile dysfunction [20–22]. DCM often exhibits a prolonged subclinical period [23, 24] making it difficult for the clinicians to diagnose the disease at the early stage. Recent studies from us and others suggest that abnormal molecular alterations from early stages of the disease form the basis for the development of structural and functional deterioration at the later stages [25–27].

### Molecular mechanisms underlying DCM

**Fig. 1:**
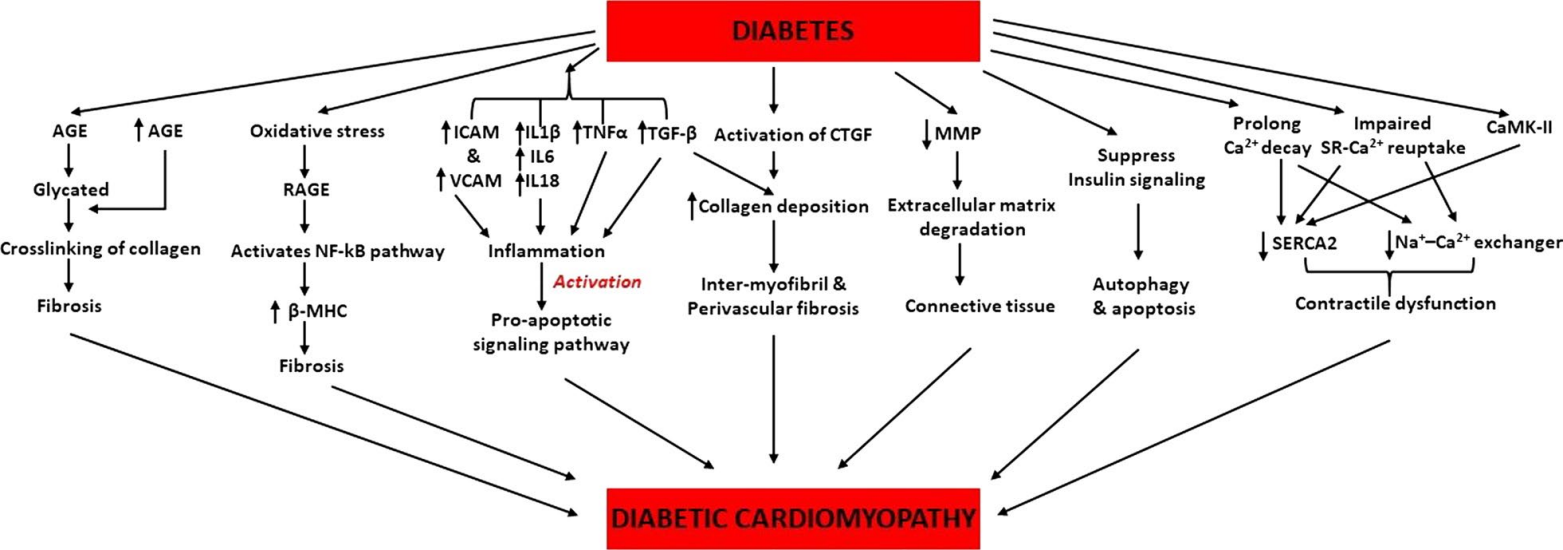
Mechanisms involved in the pathophysiology of diabetic cardiomyopathy. Schematic representation of the multiple potential mechanisms that have been implicated in the pathophysiology of diabetic cardiomyopathy. The steady increase in reports presenting novel mechanistic data on this subject expands the list of potential underlying mechanisms

Diabetes associated metabolic changes initiate a series of molecular events in the myocardium (summarized in Fig. 1). Advance glycation end products (AGE) become glycated in presence of sugar, leading to alteration in its functional properties [28]. Importantly increased the formation of AGE alters structural proteins increasing the crosslinking of collagen fibres eventually progressing to fibrosis [29]. AGEs produced its action through AGE receptors (RAGE) which are activated by oxidative stress in the diabetic myocardium [30]. Aragno et al. suggested that activation of RAGE indirectly activates nuclear factor kappa (NF-κB) signalling pathway, which may contribute to the switch towards increased expression of the β-myosin heavy chain (β-MHC) isoform in diabetic hearts [30]. Treatment with dehydroepiandrosterone in streptozotocin (STZ) induced diabetic Wistar rats, counteracts oxidative stress-induced RAGE activation, normalize NF-κB signalling pathway and β-MHC isoform shift, which are early events in DCM [31]. Diabetic myocardium also displays cross-linked AGEs on the sarcoplasmic reticulum (SR) calcium ATPase pump, which impairs SR-Ca^2+^ reuptake in myocytes [31, 32]. Inter-myofibril and perivascular fibrosis are commonly observed in DCM usually due to activation of transverse growth factor β (TGF-β) and connective tissue growth factor (CTGF) which leads to increased collagen deposition [33, 34]. Chiu et al. suggested that elevation in TGF-β may be due to activation of poly-ADP-ribose-polymerase-1 (PARP-1) [34]. On other hand, matrix-metallo-proteinase (MMP) also plays a crucial role in extracellular matrix degradation leading to increased deposition of connective tissue in the diabetic heart [35]. Activation of inflammatory cytokines also plays a key role in the pathogenesis of DCM [36–40]. Increased expression of inter-cell adhesion molecules-1 (ICAM1), vascular cell adhesion molecule-1 (VCAM1) and inflammatory cytokines like interleukin (IL) IL1β, IL6, IL18, tumour necrosis factor-α (TNFα) and TGF-β leads to myocyte inflammation in the diabetic heart [41–44]. Increased fibrosis and inflammation leads to activation of the pro-apoptotic signalling pathway [45–48]. On other hand, ob/ob mouse model of type 2 diabetes demonstrated, elevated intracellular resting Ca^2+^ concentration, prolonged intracellular resting Ca^2+^ decay, decreased sarco/endoplasmic reticulum Ca^2+^-ATPase 2a (SERCA2) activity and impaired sarcoplasmic reticulum (SR)-Ca^2+^ reuptake [49, 50]. Impairment in intracellular Ca^2+^ handling in the heart also occurred in rodent models of type 1 diabetes, involving increased resting Ca^2+^ levels, attenuated SR-Ca^2+^ release and reuptake, delayed recovery of the intracellular Ca^2+^ transient, reduced expression of SERCA-2a and the Na^+^–Ca^2+^ exchanger and compromised mitochondrial Ca^2+^ handling [51–57]. Diabetes also induces modifications of Ca^2+^/calmodulin-dependent protein kinase 2 (CaMK-II) which has a direct relation with SERCA, linked with the pathophysiology of contractile dysfunction [58, 59]. Insulin signalling is an important regulator of myocardial autophagy [60]. Mellor et al. showed increased autophagy markers such as microtubule-associated protein 1A/1B-light chain 3 and nucleoporin p62 in male C57Bl/6 mice fed with high carbohydrate diet for 12 weeks [61]. Similarly, our earlier study showed marked activation of autophagy in type-2 diabetic db/db mice and human right atrial appendage [62]. Phosphatidylinositol 3-kinase (PI3K)/Akt signalling can negatively regulate autophagy by inhibiting the mammalian target of rapamycin (mTOR) and changes in autophagy were associated with reduced PI3K/Akt signalling in insulin-resistant hearts [63, 64].

Above evidence provide a strong molecular association in the pathogenesis of the diabetic heart (summarized in Fig. 1). Our current understanding of the regulation of cardiac gene expression is at the transcriptional level, where transcriptional factors are known to govern the activation of the specific gene. However, recent studies revealed that a single gene can be controlled by multiple regulators at post-transcriptional level via microRNAs (miRs) [65, 66].

### Role of microRNAs (miRs) in DCM

MiRs have been described as the “micromanagers” of gene expression [67]. The discovery of miRs as the regulators of multiple cardiac genes has added a new mechanistic link between gene regulation in the normal and diseased heart at the post-transcriptional level. Recently, miRs have been identified as a key element involved in cardiac gene remodelling in diabetic heart [68, 69]. As these processes involve the dysregulation of multiple genes, it is reasonable to hypothesize that miRs play a key role in the pathogenesis of DCM (Table 1). Alterations in the synthesis and in the levels of specific miR have been shown to play critical roles in cardiac remodelling and the development of heart failure [70–72]. In addition, miR can affect other epigenetic changes such as alterations in histone H2A mRNA levels, histone deacetylase (HDAC) and DNA methyltransferases (DNMT), resulting in the modulation of gene expression [73]. Further, miRs can be transferred between the cells or to the distant tissue through circulation where they remain stable [74]. This ability of miRs to remain stable in the circulation makes them a promising diagnostic tool for various diseases including cardiovascular diseases. Taken together, miR may play a potential diagnostic, prognostic and therapeutic role in DCM. While miRs are involved in various pathophysiological processes of DCM, this review will focus on miRs associated with cardiac cell death and abnormal structural alteration.

**Table 1:** List of some selected miR candidates involved in the progression of DCM

Sl no	miRs	Expression pattern	Regulatory genes	Pathophysiological role	Expression pattern	Expression sight	References
1	miR-1	Downregulation Upregulation	RyR2 GATA4, NRVMs, ET1, ISO, Pim-1	Cardiac hypertrophy, apoptosis, heart failure, arrhythmia, oxidative stress	Cardiac and skeletal muscle	Transgenic mice with AKt overexpression, exercise-induced hypertrophy model in rats (neonatal rat ventricular cardiomyocytes), hypertrophic human atria and ventricles, STZ-diabetic mice, HG-exposed rat cardiomyocytes	[150] [78, 152]
2	miR-1/206	Upregulation	Pim-1, Hsp60	Cardiac apoptosis	Cardiac muscle	STZ-diabetic rat and mouse heart	[281]
3	miR-9	Downregulation	ELAVL1	Cardiac structural damage	Cardiac muscle	Human diabetic heart IHVC	[123]
4	miR-133a	Downregulation	SGK1, IGF-1R, TGF-β1	Cardiac hypertrophy and fibrosis	Cardiac and skeletal muscle	STZ-diabetic mouse heart	[145, 148]
5	miR-21	Upregulation	DUSP8	Cardiac fibrosis	Cardiac muscle	High glucose induced primary NRCFs	[153]
6	miR-29	Upregulation	MCL-1	Cardiac structural damage	Cardiac muscle	ZDF rat heart	[282]
7	miR-34a	Upregulation	BCL-2, SIRT-1	Cardiac apoptosis	Cardiac muscle	H9c2 cells	[26, 108]
8	miR-30d	Upregulation	FOXO-3a	Cardiac pyroptosis	Cardiac muscle	HFD induced rat heart	[121]
9	miR-195	Upregulation	SIRT-1, BCL-2	Cardiac apoptosis	Cardiac muscle	Db/db mice, CEC, STZ- diabetic mouse	[90]
10	miR-141	Upregulation	Slc25a3	Mitochondrial dysfunction	Cardiac muscle	STZ-diabetic mouse	[161]
11	miR-144	Downregulation	Nrf2	Cardiac apoptosis and oxidative stress	Cardiac muscle, hematopoietic cells, vein, spleen, thyroid	STZ-diabetic mouse, high glucose treated cardiomyocyte	[115]
12	miR-208a	Upregulation	GATA4, Thrap-1	Cardiac hypertrophy	Cardiac muscle	STZ-diabetic mouse	[78, 283]
13	miR-320	Upregulation	VEGF-c, IGF-1, IGF-1R, FLK-1	Cardiac apoptosis	Cardiac muscle	Human Right atrial appendage tissue, GK rat cardiomyocyte, MVEC	[81–84]
14	miR-373	Downregulation	MEF2c	Cardiac hypertrophy and oxidative stress	Cardiac muscle	High glucose induce neonatal rat ventricular cardiomyocytes, STZ- diabetic mouse heart	[150, 151]
15	miR-483-3p	Upregulation	IGF-1, BCL-2	Cardiac apoptosis	Cardiac muscle, pancreatic-βcells and adipose tissue	H9c2 cells, STZ-diabetic mouse heart	[119]
16	miR-451	Upregulation	CAB-39	Cardiac hypertrophy	Cardiac muscle	HFD induced mouse heart	[142]
17	miR-378	Downregulation Upregulation	IGF-1R	Cardiac hypertrophy Cardiac apoptosis	Cardiac muscle and muscle tissues	Rat cardiomyocytes STZ-diabetic mouse heart	[116, 118, 284]

STZ, streptozotocin; HG, high glucose; IHVC, immortalized human ventricular cardiomyocytes; NRCF, neonatal rat cardiac fibroblast; ZFD, zucker diabetic fatty rat; HFD, high fat diet; GK, Goto–Kakizaki; CEC, cardiac endothelial cells; MVEC, micro vascular endothelial cells

#### Biogenesis of miRs

**Fig. 2:**
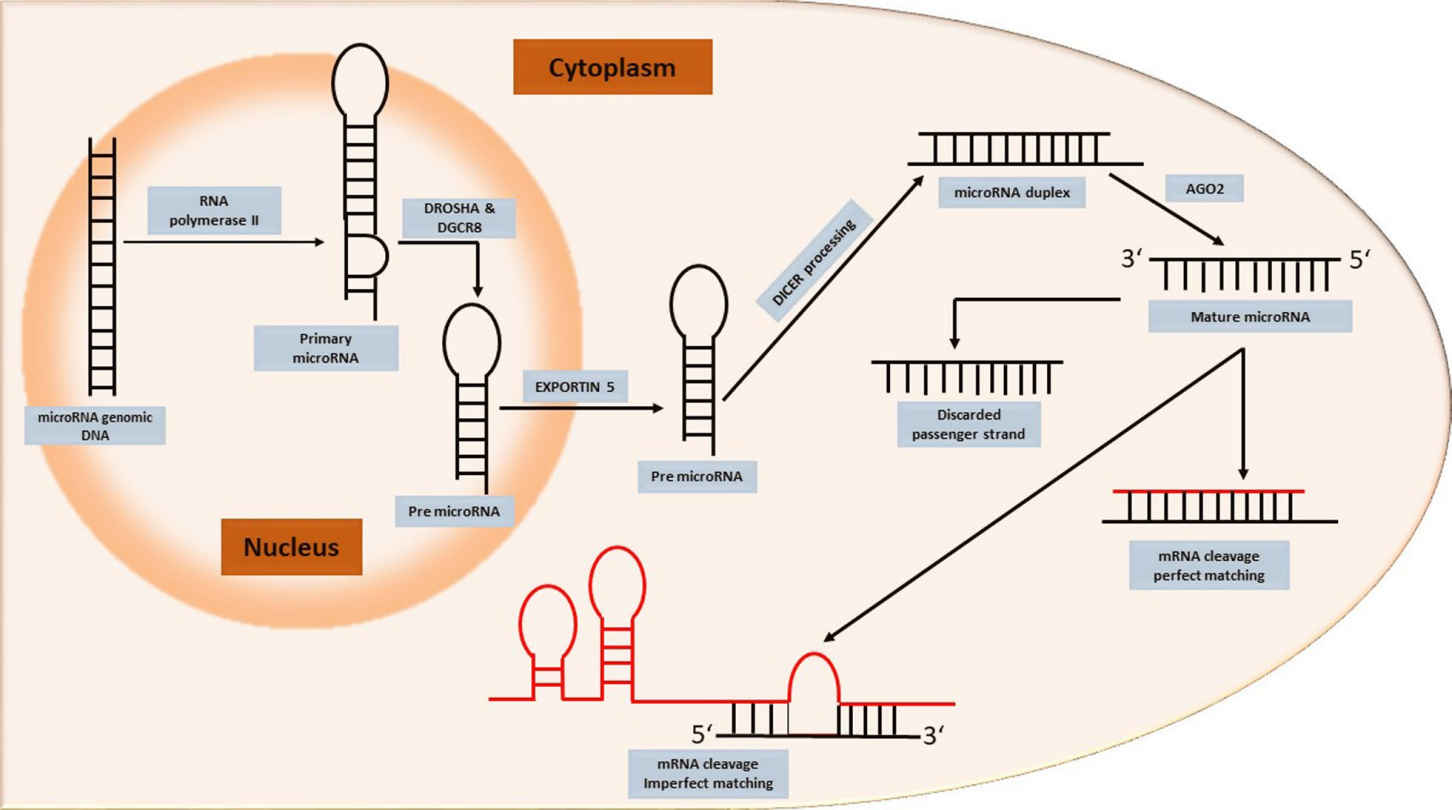
Biogenesis of miRNA. Canonical pathway of miR biogenesis in which miRs are transcribed by RNA polymerase II from intergenic, intronic, or polycistronic loci to long primary transcript, called primary miR (pri-miRNA), which consists in a stem, a terminal loop, and single-stranded RNA segments at both the 5′- and 3′-UTR sides. Microprocessor complex (Drosha and DGCR8 cofactor) cleaves the stem-loop and releases a small hairpin-shaped RNA, called precursor miRNA (pre-miRNA). Following, pre-miRNA is exported into the cytoplasm by the transport complex formed by protein Exportin 5, pre-miRNAs are cleaved by a ternary complex formed by Dicer, producing small RNA duplexes (miR–miR). Next, these are loaded onto an Argonaute 2 protein (AGO2) to form an immature RNA-induced silencing complex (RISC) or pre-RISC. AGO protein separates the two strands to generate a mature RISC effector. Finally, RISC binds the target mRNA through complementary binding of 6–8 base pairs of the miR, with a specific sequence of the target resulting in the gene silencing

MicroRNAs undergo a series of processing before maturation as discussed next. RNA polymerase-2 transcribe the miR coding genes to form several kilo-bases (kb) long primary-microRNA (pri-miR). This is cleaved further by DROSHA RNase-3 endonuclease enzyme and DGCR-8 at the basal level of stem-loop to form 70 kb long precursor-microRNAs (pre-miRs). Pre-miRs is next exported to the cytoplasm with the help of a transporter protein, Exportin-5, which is further cleaved by DICER RNase-3 endonuclease enzyme and ARGONAUTE-2 (AGO-2) protein into small interfering RNA (siRNA) like imperfect duplex comprising of one mature-miR strand and a complementary sequence. Next, the mature sequence is incorporated into ribonucleoprotein to form miR-associated silencing complex, while the complimentary sequence is degraded (Fig. 2).

#### MiRs involved in cardiac cell death

Several miRs have been shown to be associated with cardiomyocytes cell death of the diabetic heart. This section will discuss key miRs that have been demonstrated to be associated with DCM.

MiRs that belong to the miR-1 family is designated as miR-1-1 and miR-1-2 and are expressed in both cardiac and skeletal muscles [75] which shares a common bicistronic unit with miR-206. MiR-1 has been reported to control muscle differentiation by targeting Hand2 (heart and neural crest derivatives-expressed protein), a transcription factor that regulates expansion of ventricular cardiomyocytes [76]. Interestingly, the expression pattern of miR-1 is regulated by serum response factor (SRF), myocyte enhancer factor-2 (MEF2) and MyoD [66, 76, 77]. Several studies have demonstrated the important role of miR-1 in various cardiovascular diseases. Like some other miRs, miR-1 is having a dual or bidirectional role in the progression of DCM based on its expression pattern. Upregulation of miR-1, plays a pro-apoptotic role (described in this part) and the anti-hypertrophic role related to down regulation of miR-1 has been explained under next subtitle; “MiRs involved in cardiac hypertrophy, fibrosis and oxidative stress”.

The role of miR-1 in the diabetic heart has been recently demonstrated by Moore et al. which showed marked upregulation of miR-1 in type-2 mouse and human diabetic heart [78]. They further demonstrated that increased miR-1 is associated with significant downregulation of the pro-survival proto-oncogene serine/threonine-protein kinase (Pim-1) kinase, the direct protein target for miR-1 [79]. This was associated with increased cardiomyocytes apoptosis. Earlier studies by Katare et al. showed significant attenuation of diabetes-induced cardiomyocytes apoptosis following restoration of Pim-1 levels via systemic administration of Pim-1 conjugated to adeno-associated virus (AAV) [80].

MiR-320 expressed abundantly in cardiomyocytes and myocardial microvascular endothelial cells (MMVEC) [81, 82]. Wang et al. demonstrated the correlation between miR-320 expression and high glucose by co-culturing cardiomyocytes and MMEC under high glucose condition. High glucose significantly upregulated the expression of miR-320 in cardiomyocytes. Interestingly, miR-320 was then secreted into the culture medium and was uptaken by MMECs via exosomes [82]. To confirm if the intra cellular transfer of miR-320 occurs via exosomes, the authors treated the cells with exosomes inhibitor GW-4869 which reduced the uptake of miR-320 by the endothelial cells [83]. MiR-320 has the potential to target multiple angiogenetic genes such as fetal liver kinase-1 (FLK-1), vascular endothelial growth factor-c (VEGF-c), fibroblast growth factors (FGFs) and anti-apoptotic genes such as insulin like growth factor-1 (IGF-1) and insulin like growth factor-1R (IGF-1R) [82]. In support of this, an increase in miR-320 expression was associated with downregulation of IGF-1 and IGF-1R and increased apoptosis [82]. Interestingly, activation of IGF-1 and IGF-1R also exaggerated the expression of pro-angiogenic VEGF and FLK-1. Further, overexpression of miR-320 in cardiomyocytes reduced the expression of anti-apoptotic protein Bcl-2 while increasing the expression of pro-apoptotic proteins Bax and caspase-3, leading to cell death [84].

MiR-195 shares the same family and common seed region with miR-15, miR-16 and miR-497 but clustered on three separate chromosomes [85]. Several studies have confirmed the role of miR-195 in various cardiovascular and other diseases. With respect to circulating cardiovascular miRs, miR-195 has gained considerable interest as the potential diagnostic biomarker for the diagnosis of cardiovascular diseases [86]. Diabetes has been reported to regulate miR-195 expression in animal models. Chen et al. demonstrated reduced expression of miR-195 in the kidney of STZ-induced type 1 diabetic mice [87] whereas, Herrera et al. and Mortuza et al. showed upregulation of miR-195 in liver and retina of type 2 diabetic rat [88] and in STZ-induced diabetic rats [89], respectively. Those studies evidenced that there is a tissue-dependent differential expression of miR-195 in diabetes. Zheng et al. demonstrate that both type 1 and type 2 diabetes promote expression of miR-195 in the heart [90] leading to apoptosis and hypertrophy in cardiomyocytes under stress [91, 92]. Importantly, silencing of miR-195 reduced apoptosis, oxidative stress and hypertrophy, thereby improving the myocardial function. These evidence indicates that miR-195 makes a significant contribution to DCM.

MiR-30c is differentially expressed in the heart during the progression towards heart failure [93]. MiR-30c and miR-181a were identified to target genes of p53 and p21 which are key regulators of cardiomyocyte hypertrophy but also apoptosis. Both p53 and p21 were found to be upregulated by miR30c and miR-181a in DCM [94]. Silencing miR-30c resulted in a strong apoptotic phenotype in cell culture, suggesting that miR-30c is anti-apoptotic [95]. A decreased expression of miR-30c and miR-181a has been earlier reported in myocardial infarction, dilated cardiomyopathy, left ventricular hypertrophy, cardiac fibrosis and apoptosis and, heart failure [96–103]. They act by directly targeting the pro-hypertrophic and apoptotic regulators p53 and p21. Further, Raut et al. observed a significant decrease in the number of apoptotic cells in high glucose-treated H9c2 cells overexpressed with either miR-30c or miR-181a mimics as compared to non-transfected HG-treated cells suggesting a possible role for miR-30c and miR-181a in DCM [94]. Moreover, Chen et al. found that the depletion of miR-30c and induction of BECN1 (beclin-1; a protein encoded by BECN1 gene) enhanced autophagy in diabetic (db/db mouse) hearts and in cardiomyocytes treated with the fatty acid palmitate [104]. This finding suggests a possible regulatory association of miR-30c, autophagy, and DCM.

The miR-34 family consists of three members; miR34a on human chromosome 1p36, and miR-34b and miR-34c co-transcribed on human chromosome 11q23 [105]. Among the three isoforms, miR-34a is abundantly expressed in cardiomyocytes [106, 107]. MiR-34a has been associated with the regulation of several proteins like Bcl-2 [108], Akt (serine/threonine-specific protein kinase) [109], phosphatase 1 nuclear targeting subunit (Pnuts) [106], and sirtuin 1 (SIRT1) [110], all of which have actions relevant to the diabetic heart. SIRT1 plays a major role in senescence in diabetic heart [111]. In a recent study, we showed a marked upregulation of circulating miR-34a in type-2 diabetic individuals without any history of cardiovascular disease from the early stages of diabetes. This was further supported by upregulation of miR-34a in the diabetic human heart and in human cardiomyocytes cultured under high glucose conditions [26] and this was associated with downregulation of SIRT-1. Importantly, inhibition of miR-34a activity in high glucose cultured cardiomyocytes restored the levels of SIRT-1 [26]. Similarly, another study showed an elevated expression of miR-34a in pre-diabetic and newly diagnosed type 2 diabetic individuals [112]. Using in vitro cultured cardiomyocytes, Zhao et al. demonstrated marked upregulation of miR-34a levels by high glucose which was associated with downregulation of anti-apoptotic Bcl-2 and increased cardiomyocyte apoptosis [108]. They also demonstrated a significant decrease in Bcl-2 expression following treatment with miR-34a mimic, whereas treatment with anti-miR-34a increased the Bcl-2 expression and reduced high glucose-induced cardiomyocyte apoptosis [108].

MiR-144 is not cardiac-specific. This miR is abundantly expressed in hematopoietic cells, vein, spleen, thyroid and heart [69, 113]. Recent studies have documented that nuclear factor-erythroid 2-related factor 2 (Nrf2) was repressed by miR-144 in K562 cells [114] and cerebro-microvascular endothelial cells [114]. Yu et al. demonstrated that Nrf2 was a direct target of miR-144 in adult cardiomyocytes. In addition, repression of miR-144 prevented myocardial apoptosis by mitigation of oxidative stress in STZ-challenged diabetic mice [115]. Treatment with miR-144 mimics aggravates high glucose-induced oxidative stress and apoptosis which was reversed by treatment with the Nrf2 activator, indicating that miR-144 is a direct regulator of Nrf2 [115]. This was also replicated in vivo where treating diabetic mice with anti-miR-144 enhanced the expression of Nrf2 in hearts and reduced apoptosis.

Mir-378 family with most of the family members are abundantly expressed in muscle tissues and the myocardium. MiR-378 is a key initiator of apoptosis, which is significantly overexpressed in the diabetic heart and intensive glycemic control was unable to revert these changes [116]. Knezevic et al. suggested that overexpressed miR-378 reduced the expression level of endogenous anti-apoptotic IGF-1R [82, 117], whereas treatment with anti-miR-378 upregulated the endogenous level of IGF-1R thereby reducing the cardiomyocytes apoptosis [118]. Moreover, miR-483-3p is abundantly found in pancreatic-βcells, adipose tissue including cardiomyocytes. Interestingly, upregulation of miR-483-3p in diabetic hearts and cardiomyocytes under hyperglycemia mimicking condition promotes apoptosis by downregulating the expression of the anti-apoptotic protein Bcl-2 and IGF-1 respectively. Hence, miR-483-3p enhance cell death by miR-483-3p-Bcl2 and IGF pathway [119]. On other hand, miR-195, expressed mostly in adipocytes, liver and myocardium; especially in cardiomyocytes and MMVEC plays a crucial role in post diabetic cardiac complication. Upregulation of miR-195 has been demonstrated to increase apoptosis in cardiomyocytes and endothelial cells in vitro and miR-195 inhibition prevented apoptosis in cardiac endothelial cells in response to non-esterified fatty acid (NEFA) such as palmitate [90].

MiR-30d and miR-30a belong to miR-30 family, expressed abundantly in cardiomyocytes and adipose tissue respectively [120, 121]. Some family members including miR-30c, miR-30d, and miR-30e acts as a potential biomarker for ovarian cancer. Li et al. demonstrated that upregulation of miR-30d in high glucose-treated cardiomyocytes and in the diabetic heart suppress fork-head box-O3 (FOXO-3a), a crucial regulator of diverse cellular activities, such as cell cycle arrest, oxidative scavenging, cell proliferation, survival and death and apoptosis repressor with caspase recruitment domain (ARC) expression, inflammatory molecules and promote pyroptosis, which is an inflammatory form of programmed cell death [121]. Foxo3a is involved in the inhibition of cell death and promotion of cellular growth and cardiac remodelling in diabetes [122]. More importantly, overexpression of miR-30d resulted in increased levels of caspase-1, interleukin-1β (IL-1β) and IL-18, whereas knockdown of miR-30d decreased inflammatory cell death in cardiomyocytes indicating anti-miR-30d as a strategy for the prevention of cardiac pyroptosis.

MiR-9 family which includes miR-9a1, miR-9a2, miR-9b and miR-9c are mainly expressed in heart muscle and brain. The role of miR-9 in high glucose-induced inflammation or cardiac pyroptosis and its effect on cardiovascular diseases is very limited. Jeyabal et al. demonstrated that elevation of ELAV-like protein 1 (ELAVL1) is dependent on the downregulation miR-9 in the human diabetic heart [123]. Inhibition of miR-9 resulted in an elevation of ELAVL1 protein expression along with caspase-1 and IL-1b expression, while miR-9 mimic transfection reduced ELAVL1, caspase-1 and IL-1b expression. Dual luciferase reporter assay validated that ELAVL1 is a direct target of miR-9 in cardiomyocytes.

MiRs expression in diabetic vascular complication is regulated by diverse factors. Jansen et al. demonstrated direct effects of diabetes mellitus in altering the expression of vascular miRs in circulating micro particles [124]. Moreover, hyperglycemic individuals have elevated levels of circulating micro particles, which might be actively involved in the progression of vascular dysfunction in diabetic conditions [124]. Micro particles are vesicular structures shed from endothelial or circulating blood cells, after activation or apoptosis, and can be considered as markers of vascular damage. They are one of the major transport vehicles of miRs and contribute to the vascular homeostasis [125–127]. Reports show an important role for endothelium-enriched miR-126 in vascular homeostasis and angiogenesis [128, 129]. Through micro particles transporter system, miR-126-3p promotes recipient vascular endothelial cell repair, however micro particles obtained in hyperglycemic conditions show reduced regenerative capacity both in vivo and in vitro [130]. We along with others have demonstrated reduced expression of miR-126 in type-2 diabetic individuals who were otherwise healthy confirming the loss of vascular protection in the early phases of glucose intolerance [129, 131, 132]. More recently, Giannella et al. demonstrated the abnormality in circulating micro particles (CD62E^+^) and its contents (including miR-126) in subjects with different degrees of glucose tolerance [133]. Results showed that a significant decrease in the expression of miR-126 in type 2 hyperglycemic patients in the micro particles was associated with increased apoptosis in endothelial cells.

Taken together, miRs may regulate different pro-apoptotic and anti-apoptotic signalling pathways in endorsing or shielding against DCM. Evidence-based study suggests that alteration of certain miRs may impact series of signal transduction pathway leading to apoptosis, which works synergistically in the progression of DCM. The major miR candidates involved in cell death are summarized in Table 1.

#### MiRs involved in cardiac hypertrophy, fibrosis and oxidative stress

Diabetes eventually induces oxidative stress and structural damage such as hypertrophy and fibrosis, which are typically the compensatory responses in cardiomyopathy. Several miRs including miR-1, miR-133a, miR-373, miR-378, miR-23b, miR-181, miR-30c, miR-208, miR-195 and miR-451 have been shown to be associated with these events.

MiR-208a is a cardiac-specific miR which is encoded within an intron 29 of Myh6 gene encoded with myosin heavy chain-α (MHCα). The MHCα along with MHCβ isoforms are major contractile proteins of cardiomyocytes which differ in their ability to convert ATP to mechanical work at different rates that affect the contractility of the cardiac sarcomeres [134, 135]. Increased expression of MHCβ is a common feature of cardiac hypertrophy and heart failure that reduces contractile performance [136–138]. Interestingly, the hypertrophic growth induced by miR-208a is only accompanied by increased MHCβ expression in a subset of hypertrophied cardiomyocytes. In contrast, deletion of miR-208a resulted in decreased MHCβ expression in the adult heart [139]. Upregulated MHCβ expression in miR-208a transgenic mouse hearts is associated with regional fibrosis [140]. MiR-208a post transcriptionally represses the expression of Thrap-1, a component of the thyroid hormone nuclear receptor complex. Studies have also shown that increasing the level of miR-208a in transgenic mouse hearts reduced Thrap-1 levels and induced hypertrophic growth, thereby providing a link between the action of miR-208a and thyroid hormone in cardiac hypertrophy. Further, GATA-4 the key cardiac transcription factor is also directly targeted by miR-208a, suggesting the crucial role for miR-208a cardiac transcription.

As discussed earlier on the dual role of miR-1, Zaglia et al. demonstrated that downregulation of mitochondrial calcium uniporter (MCU) occurs during physiologic and pathologic remodelling in human hearts. MCU play a critical role in the response to β-adrenoreceptor stimulation occurring during acute exercise. Zaglia et al. showed that miR-1 as a direct target for MCU inhibiting its translation, thereby affecting the capacity of the mitochondrial Ca^2+^ uptake machinery, thereby playing a crucial role in cardiomyocytes hypertrophy [141].

MiR-195 found abundantly in the blood of individuals with or without metabolic syndrome [27]. Though this miR has the pro-apoptotic effect, it also plays a pivotal role in the progression of hypertrophy [90]. Zheng et al. showed that Bcl-2 and SIRT1 expression was decreased in both STZ-induced type 1 and/db type 2 diabetic mice heart, in response to elevated expression of miR-195, which was restored following anti-miR administration [90]. Similarly, Kuwabara et al. showed that upregulation of miR-451 in type 2 diabetic (C57BL/6 transgenic) mice and neonatal rat cardiomyocytes in response to the excess supply of saturated fatty acids playing a crucial role in the development of DCM [142]. Calcium-binding protein 39 (CAB39) is an upstream kinase of AMP-activated protein kinase (AMPK), which is a direct target of miR-451. Eventually, overexpression of CAB39 rescued cardiac hypertrophy.

MiR-133 and miR-1 are reported to be expressed specifically in cardiac and skeletal muscles [90]. MiR-133 family includes miR-133a-1, miR-133a-2 (both of them expressed in cardiac and skeletal muscles) and miR-133b (specifically expressed in cardiac muscle). While all these miRs share a common bicistronic unit with miR-1 and miR-206, they are also expressed as separate transcripts and exhibit different phenotypes [143]. Care et al. was the first to demonstrate that among the 3 major direct targets for miR-133 (RhoA a GDP-GTP exchange protein), Cdc42 (signal transduction kinase) and Nelf-A/WHSC2 (nuclear factor), RhoA and Cdc42 have a distinct role in regulating cardiac hypertrophy [144]. In support of the above findings, another study showed that reduced expression of miR-133a facilitates hypertrophic progression in STZ induced type 1 diabetes model [145]. On other hand, miR-133a is also a direct target of myocyte enhancer factor-2C (MEF-2C) which is a key transcription factor for myocardial hypertrophy and fibrosis through activation of p300 gene [146, 147]. Reduced expression of miR-133a leads to upregulation of serum and glucocorticoid regulated kinase-1 (SGK-1) and IGF-1R resulting in the activation of MEF-2C [147]. Moreover, miR-133a also have an effect on fibrosis TGF-β1 pathway [148]. MiR-133a can also regulate calcineurin-nuclear factor in activated T cells c4 (NFATc4) signalling and DNA methyltransferases-1 (DNMTs-1)-3a, and DNMTs-1-3b, all of which are changed in diabetic hearts and have been shown to be related with a hypertrophic response and cardiac remodelling [145, 147, 149]. Furthermore, Yildirim et al. demonstrated that RyR2 located in sarcoplasmic reticulum is a target protein for miR-1 in the diabetic heart, which exerts a vital role in Ca^2+^ transport and cardiomyocyte contractility [150].

MiR-373 is another known anti-hypertrophic miR shown to have a correlation with p38, a member of mitogen-activated protein kinase (MAPK) signalling pathway. MiR-373 is transcriptionally regulated by p38 MAPK and miR-373 can protect against glucose-induced hypertrophy by repressing the MEF2C (hypertrophic protein) [151]. Similarly, in addition to its pro-apoptotic property, miR-1 has been shown to exhibit an anti-hypertrophic effect on cardiomyocytes by negatively regulating calmodulin and the Nuclear factor of activated T-cells (NFAT) signalling [152].

Several other miRs have been suggested to play a critical role in cardiac fibrosis, including miR-21, miR-125b, miR-150, miR-199a, miR-29b, miR-30a, miR-142-3p and miR-700. Liu et al. demonstrated that miR-21 is markedly overexpressed in cardiac fibroblasts under high glucose treatment, leading to the elevation in collagen synthesis and phosphorylated p38 MAPK [153]. Inhibition of miR-21 reduced fibrosis by preventing the activation of p38 signalling pathway [153]. MiR-142-3p and miR-700 have been shown to regulate cardiac fibrosis by modulating TGF-β_3_ and collagen, type I, alpha 1 (Col-1A-1) in the heart which demonstrated the crucial role of these two miRs in DCM [147]. Disorganization of cardiomyocytes and myofibril bundles lead to cardiac structural impairments.

The miR-15 family consists of six highly conserved miRs (miR-15a, miR-15b, miR-16, miR-195, miR-497 and miR-322), which are abundantly expressed in cardiomyocytes [154–156]. We recently demonstrated marked downregulation of both miR-15a and-15b and in type 2 human diabetic myocardium. This was associated with marked upregulation of pro-fibrotic transforming growth factor-β receptor-1 (TGF-βR1) and connective tissue growth factor (CTGF), the known protein targets for miR-15a/b [154, 157]. Moreover, using type 2 diabetic db/db mice we confirmed that downregulation of both miR-15a/b starts from the early stage of diabetes. Further, in vitro therapeutic modulation of both these miRs markedly reduced the myofibroblast differentiation of human cardiac fibroblasts [157].

MiR-29 family involves a cluster of three precursors, with miR-29a and miR-29b1 being transcribed from chromosome-7, and miR-29b2, which has an identical sequence to miR-29b1, as well as miR-29c being transcribed from chromosome-1 [158] and the clusters of miR-29 has been transcribed as polycistronic units [72, 159]. Zhang et al. demonstrated that in vitro knockdown of miR-29b in cardiac fibroblasts enhanced fibrosis while overexpression prevented this effect thereby suggesting a protective role of miR-29b in cardiac fibrosis in response to AngII [160]. This was further demonstrated in vivo where overexpression of miR-29b prevented the AngII-mediated cardiac fibrosis and cardiac dysfunction.

Mitochondria are the major source for the production of reactive oxygen species (ROS) in living cells. Elevated glucose levels can lead to mitochondrial ROS production, resulting in the damage of cellular components. Dysregulation of certain miRs including miR-144, miR-1, miR-133, miR-499, miR-195, miR-34a, miR-141, miR-200a, miR200c, miR-19b, miR27a, miR-125b, miR-155 miR-146a, miR-210, miR-373 and miR-221 have been associated with oxidative stress [115, 116, 161]. Yildirim et al. showed that miR-499, miR-1 and miR-133 are unusually repressed in high glucose-treated cardiomyocytes, while treatment with the antioxidant *N*-acetylcysteine restored the expression [150]. On other hand, downregulation of miR-373 in DCM was caused by high glucose-induced oxidative stress in the heart [151]. Another study showed that high glucose treatment elevates ROS production in response to decreased miR-144 expression by targeting nuclear factor-erythroid 2-related factor-2 (Nrf-2) which is a central mediator of cellular response to oxidative stress [147]. Likewise, among the miR-200 family which consist of miR-200a, miR-200b, miR-200c, miR-141, and miR-429 [162], miR-200a, miR-200c and miR-141 have been demonstrated to play a crucial role in oxidative-stress dependent endothelial dysfunction in diabetes and obesity. p38α, a mitogen-activated protein kinase (MAPK) is an oxidative stress sensor has been shown to regulate miR-200a and miR-141 expression pattern [163, 164]. Moreover, MiR-185, located in the 22q11.2 gene locus [165], is generally regarded as a regulator involved in the biological processes of carcinoma cells and neurological disorders [166, 167]. Interestingly, a recent study by La Sala et al. demonstrated upregulation of miR-185 in human umbilical vein endothelial cells (HUVECs) that were exposed to oscillating levels of high glucose. They further showed that upregulated miR-185 contributes to increased oxidative stress in HUVECs exposed to high glucose [168].

Taken together, different miRs may regulate different mechanisms and signalling pathways in promoting or protecting against DCM and that miRs could be a novel therapeutic strategy to overcome the deleterious effect of diabetes on the heart. Role of major miR candidates involved in DCM is summarized in Table 1.

### Techniques involved in modulation of miRs

As discussed above miRs form an integral part of several disease processes including the evolution of DCM providing a novel therapeutic approach for the treatment of DCM. While the therapeutic potential of miRs is promising, it does come with several hurdles such as stability and poor efficiency following delivery. The following section will discuss the established techniques involved in the modulation of miRs.

#### Designing of therapeutic miRs

The goal of miR replacement therapy using synthetic miRs or miR-mimics is to achieve the same biological functions as the endogenous miRs. Therefore, the synthetic miRs and anti-miRs should possess the ability to adhere to RNA-induced silencing complex (RISC). The double-stranded miR containing both guide and passenger strands was more potent than the single-stranded [169, 170] to enable the appropriate formation of RISC. Therefore, designing miR-mimics with a double-stranded structure has become the path of therapeutic development. Synthetic miRs precursors with longer sequences have also been proposed as therapeutic agents [171]. Since pri-miRs undergoes processing inside the nucleus to form mature miRs, different strategies are required for the delivery of different types of miR-mimics and anti-miRs to their target domain [172]. Due to limited sequence variation of miRs, chemical modification is the major approach to tackle this problem.

#### Structural modification of miRs

RNAs are extremely fragile to serum nucleases enzyme but double-stranded RNA is quite resistant to nuclease degradation than single-stranded RNA. Therefore modifications of the RNA duplexes can minimize immunogenicity and reduce off-target effects [173]. A number of factors like position and type of modification need to be considered for the successful modification to make miRs well-matched with the endogenous silencing pathway [174]. The majority of this class of oligonucleotides function via a steric block mechanism, in which miRs function is inhibited by strong hybridization with exogenously introduced anti-miRs in order to block RISC loading [175]. Anti-miRs may also be designed to trigger miR cleavage by RNase-H although this approach is less common [176]. A typical anti-miR is a perfect complement to the endogenous target miR which has chemical modifications to enhance nuclease stability and target binding affinity [176]. The central position of the guide strand mirror and the 5′ phosphate and 5′ proximal part are the significant part of the RNA duplex for the interaction with the RISC [177, 178] (Fig. 2). By contrast, chemical modifications at the entire passenger strand and the 3′ proximal part and 3′ projection of the guide strand would have the least influence on the specificity and function of the RNA [179]. The major types of chemical modifications that are being investigated in the preclinical setting are locked nucleic acid modification, ribose-2′-OH modification and phosphorothioate (PS) modification (summarized in Table 2).

**Table 2:** List of some selected miR candidates using chemical modification and their delivery

Target miRs	miR modification	Delivery of therapeutic miRs	Pathophysiological role	Model system	References
miR-16, miR-122, miR-192, miR-194	2′-*O*-Me	Lipid-based	Fibrosis	Mice	[205]
miR-133	2′-*O*-Me 2′-*O*-Me	Lipid-based Lentivirus	Hypertrophy Metabolic disturbance	Mice Cardiomyocytes	[144] [244]
miR-199b	2′-*O*-Me	Lipid-based	Fibrosis and functional impairment	Mice	[285]
miR-34a	LNA+PS LNA	Direct and lipid-based Lentivirus	Myocardial infarction Contractile dysfunction	Mice and cardiomyocytes C57BL/6 mice and cardiomyocytes	[187] [106]
miR-1	LNA	Direct and lipid-based	Oxidative stress	C57BL/6 mice and cardiomyocytes	[188]
miR-208	2′-*O*-Me LNA	Lipid-based Direct	Hypertrophy Heart failure	Mice Rat	[139] [186]
miR-328	2′-*O*-Me	Lipid-based	Atrial fibrillation	Mice	[242]
miR-320	2′-*O*-Me 2′-*O*-Me 2′-*O*-Me	Lipid-based Lipid-based Lentivirus	Myocardial infarction Impaired angiogenesis Myocardial infarction	Mice MMVEC Rat and cardiomyocytes	[286] [82] [81]
miR-98	2′-*O*-Me	Adenovirus	Hypertrophy	Ventricular cardiomyocytes	[240]
miR-328	LNA	Adenovirus	Atrial fibrillation	C57BL/6 mice	[242]
miR-590 and miR-199a	2′-*O*-Me	AAV and lipid-based	Cardiac functional impairment	Wistar rats and CD1 mice Adult ventricular cardiomyocytes	[251]
miR-30c	2′-*O*-Me	AAV	Impaired autophagy	H9c2 and HEK293 cell db/db mice	[104]
miR-21	2′-*O*-Me, 2′-*O*-F/MOE and LNA LNA+PS	Lipid-based and direct Direct	Fibrosis and functional impairment Fibrosis and cardiac remodeling	Mice Mice	[287] [185]
miR-322	2′-*O*-Me	Lentivirus	Cardiomyocyte apoptosis	Cardiomyocytes	[258]
miR-137	2′-*O*-Me	Lentivirus	Cardiomyocyte apoptosis	Cardiomyocytes	[259]

*O*-Me, *O*-methyl; LNA, locked nucleic acid; MMVEC, myocardial microvascular endothelial cells; F/MOE, fluro/methoxyethyl; AAV, adeno-associated virus; PS, phosphorothioate

#### Locked nucleic acid (LNA) modifications

**Fig. 3:**
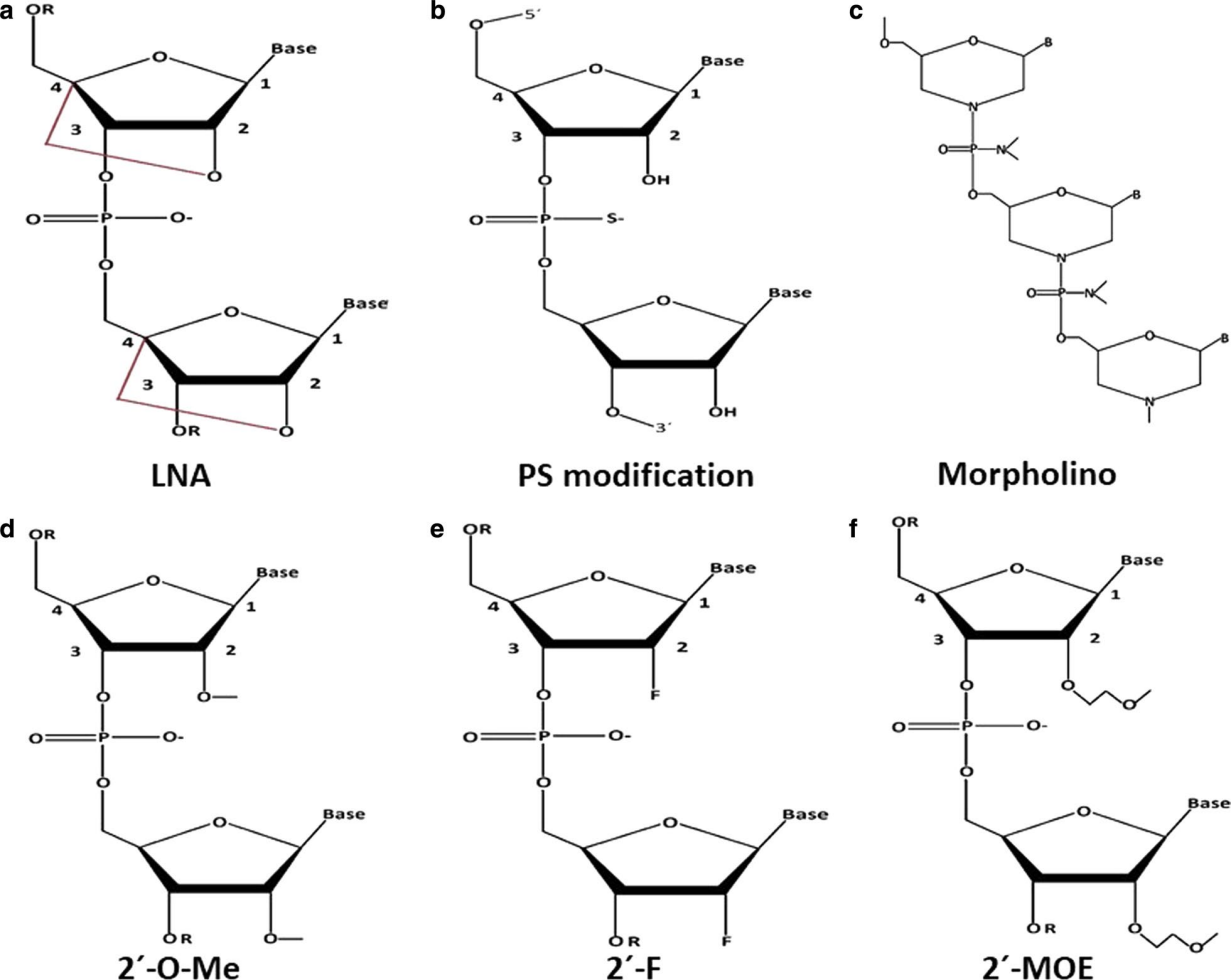
Chemical modifications of miRNA oligonucleotides. Structures of chemically modification. **a** Structures of the most commonly used chemical modifications in oligonucleotide chemistry; locked nucleic acid (LNA) is a bicyclic RNA analogue in which the ribose is locked in a C3′-*endo* conformation by introduction of a 2′-*O*,4′-C methylene bridge, **b** the nonbridging phosphate atom is replaced with a sulfur atom to give a phosphorothioate (PS) modification, **c** six-membered morpholine ring replaces the sugar moiety in morpholino oligomers. **d** In the ribose 2′-OH group modification: the 2′-OH group is modified with 2′-*O*-methyl (2′-*O*-Me), **e** 2′-OH group is modified with 2′-fluoro (2′-F), **f** 2′-OH group is modified with 2′-*O*-methoxyethyl (2′-MOE)

LNA is a modified RNA nucleotide and often referred to as inaccessible RNA. These are a class of nucleic acid analogues in which the ribose ring is “locked” by a methylene bridge connecting to an oxygen and a carbon atom in 2′ and 4′ position respectively (Fig. 3a). The bridge attaches the 3′-endo confirmation in the form of A-form duplex (possible double helical structure) [180]. LNA nucleotides contain the common nucleobases that appear in DNA and RNA and are able to form base pairs according to standard Watson–Crick base pairing rules. However, by “locking” the molecule with the methylene bridge the LNA is constrained in the ideal conformation for Watson–Crick binding. When incorporated into a DNA or RNA oligonucleotide, LNA, therefore, makes the pairing with a complementary nucleotide strand more rapidly, thereby increasing the stability of the resulting duplex. This modification improves RNA duplex stability by increasing its resistance to nuclease degradation [181, 182]. In order to be efficient, synthetic miRs must be designed in such a way that it helps the selection of the projected guide strand by the RISC to minimize off-target effects. LNA modification can avoid this type of off-target effect as the modification of the passenger strand at the 5′ end precludes its incorporation into the RISC [181]. LNA units impart the most impressive duplex stability effects of the available chemical modifications, with stabilizations of 5.6C per insert [183]. The strong binding properties of LNA make them particularly useful in anti-miR applications, where short sequences can be necessary for miR specificity, and LNAs are excellent antisense-oligos modifications when used in gapmer-constructs (contains a central block of deoxynucleotides sufficient to induce RNase-H cleavage flanked by blocks of 2′-*O*-methyl modified ribonucleotides that protect the internal block from nuclease degradation) [184]. Moreover, LNA modification, in general, reduces the immune response by preventing the immunogenic sequence-motifs without affecting its silencing activity [181].

Recent studies demonstrated that LNA induced oligonucleotides are effective in inducing potent and sustained silencing of cardio-enriched miRs [185, 186] and importantly the route of administration of LNA for cardiac targeting is flexible. Hullinger et al. demonstrated that a single injection of a low amount of LNA was able to exhibit highly effective anti-miR activity in the acute situation like ischemic injury [156]. Due to the systemic injection, multiple doses are possible which in fact produce the sustained effect. In another study, Bemardo et al. demonstrated that inhibition of miR-34a through LNA induced anti-miR-34a to prevent deterioration of cardiac function over an 8-week period in a moderate transverse aortic constriction (TAC) induced hypertrophic cardiomyopathy mouse model [187]. On other hand, Wang et al. demonstrated that LNA-induced-anti-miR-1 markedly repress the expression of ROS in the cardiomyocytes of neonatal Wistar rats and transgenic mice by decreasing the expression of miR-1 [188]. Similarly, another study demonstrated the beneficial role of LNA-induced-anti-miR-34a in mice with preexisting pathological cardiac remodelling and dysfunction due to myocardial infarction (MI) [107]. Moreover, inhibition of miR-143 using the LNA inhibitor for miR-143 protected against the detrimental effects of DCM by inhibition of Akt signalling activity [189]. Further, subcutaneous delivery of LNA-induced-anti-miR-208 in a mouse can effectively elevate myosin-heavy-chain which is a target of miR-208 and provides a protection during heart failure [186]. Furthermore, inhibition of miRs by using short-seed-targeting LNA (tiny LNA or LNA modified 8-mer PS) [190] which has the high binding affinity towards the complementary miR-seed region enable specific and concentration-dependent inhibition of entire miR-seed families in the in vitro cultured cells with concomitant restoration of their direct targets [190].

#### Phosphorothioate (PS) modification

**Fig. 4:**
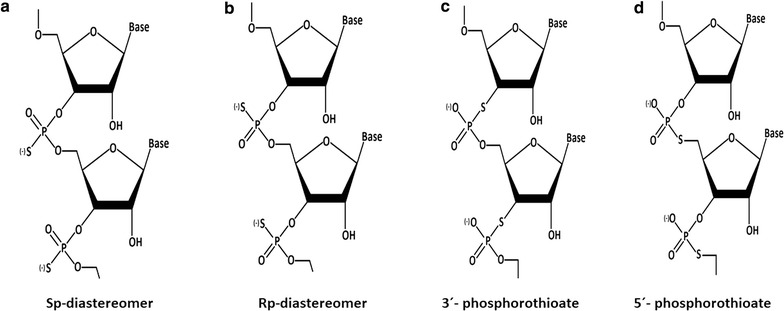
Isomeric configuration phosphorothioate modification. Formation of diastereomers by phosphorothioate (PS) backbone modifications. **a**, **b** Chiral arrangements of Sp and Rp diastereomers. **c**, **d** Formation of 3′-phosphorothioate and 5′-phosphorothioate inter-nucleotide linkage from Sp and Rp diastereomers

The phosphorothioate (PS) modification is also termed as backbone modification of oligonucleotides. Backbone modifications are mainly used to increase the stability of nucleic acids against nuclease resistance by substituting the phosphodiester linkages with other linkage. In this process of modification, the non-bridging oxygen atom is replaced by a sulfur atom in the phosphate group (Fig. 3b) [191] which is referred to as phosphorothioate (PS) backbone modifications. Phosphorous itself has four non-identical ligands which can be arranged chirally (the Greek word for hand). During chemical synthesis, two compounds forms, called diastereomers (Sp and Rp), with different configurations around phosphorus atom (Fig. 4a, b). This two diastereomers can be expected to interact differently with enzymes [192] thereby providing a variation in nuclease resistance [193, 194]. In addition, sulfur is a “soft” atom that coordinates preferentially with “soft” metal ions, whereas oxygen is a “hard” atom that coordinates to “hard” metal ions. Therefore, use of PSs also permits the identification of metal-ion binding sites in the biological systems. The PS-inter-nucleotide linkage is considerably more stable than the phosphodiester bond toward nucleases, and this feature makes it useful for cell culture and in vivo studies [193]. This two diastereomers (Sp and Rp), helps in the formation of 3′-phosphorothioate and 5′-phosphorothioate inter-nucleotide linkage (Fig. 4c, d).

Similarly, the non-bridging oxygen atom can also be replaced by using morpholino oligomers, in which a six-membered morpholine ring replaces the sugar moiety (Fig. 3c) [195]. Morpholine oligomers are uncharged, sequence-specific, non-toxic resistant to nucleases degradation which provides a slight increase in binding affinity towards both prior-miRs and mature-miRs [195, 196].

Apart from nuclease resistance, PS backbone modifications also enhance binding to plasma proteins, leading to reduced clearance by glomerular filtration and urinary excretion. Thus, PS-modified oligonucleotides exhibit markedly improved pharmacokinetic properties, facilitating their delivery into many peripheral tissues in vivo [169]. Several studies have evaluated the efficacy of chemical modification in anti-miRs. Davis et al. demonstrated a relationship between binding affinity and the potency of fully PS modified anti-miRs, having the highest melting temperature (T_m_) and potency [197]. Using luciferase assay they concluded that, LNA/2′-methoxyethyl (2′-MOE) mix-anti-miR and 2′-F with PS modification having the most potent inhibitory effect on miR-21in comparison to unmodified 2′-*O*-methyl (2′-*O*-Me). Similarly, Lennox and Behlke demonstrated LNA/2′-*O*-methyl (2′-*O*-Me) mix with PS (end or complete) modification, which is having 10 times more potent inhibitory effect on miR-21 in HeLa cells than a uniform 2′-*O*-Me modified anti-miR [198]. Care et al. showed, in vivo inhibition of miR-133 in mice heart were sufficient to induce significant hypertrophic growth [144]. Similarly, Van Rooij et al. demonstrated, down-regulation of miR-29 with anti-miRs in vitro and in vivo induces the expression of collagens, whereas over-expression of miR-29 with mimics in fibroblasts reduces collagen expression [199].

#### Ribose-2′-OH modification

Modification of the ribose 2′-OH group is another modification in RNA duplex design to enhance its stability [200]. Apart from enhancing nuclease stability, ribose 2′-OH modifications also reduce immune activation of the RNA duplex [201]. Ribose 2′-OH group modification of 2′-*O*-methyl (2′-*O*-Me), 2′-fluoro (2′-F) and 2′-methoxyethyl (2′-*O*-MOE) substitution in 2′-the position has been demonstrated to effectively improve the stability of RNA duplex (Fig. 3c–e) [202]. Though 2′-OH modification provides stability and reduces immune activation, most of the time modifications arranged in combination with LNA or PS modification or sometimes altogether. As for example, Davis et al. identified a chimeric 2′-F/MOE as a potent inhibitor of miR activity in vivo which does not involve degradation of the miRs [203]. This speciality of 2′-F/MOE modification is crucial for interpreting miR inhibition studies. In support of this, 2′-MOE-modified anti-miR-21 and a cholesterol-conjugated anti-miR-122 showed substantial binding efficiency and superior effect in cultured Hela cells and in vivo respectively [197, 204]. Further, Krützfeldt et al. demonstrated that administration of 2′-*O*-Me-anti-miRs against miR-16, miR-122, miR-192 and miR-194 resulted in a marked reduction of the corresponding miR levels, having a significant role in cardiac hypertrophy and fibrosis [205]. In mice treated with antagomir-16, miR-16 was efficiently silenced in all tissues tested including heart except brain [205]. Similarly, Care et al. showed that inhibition of miR-133 leads to hypertrophy using a similar modification of the corresponding anti-miR in vivo [144].

Taken together, each of the chemical modifications has significant advantage with few drawbacks. Hence, the selection of chemical modification is a vital step in the modulation of miRs. Moreover, to get best possible results, it is sometimes necessary to use more than one modification or simultaneously all the modifications at a time based on the model system used. Among all the modifications discussed, LNA is still the best choice for chemical modification of miR which has been successfully tested in several in vitro and in vivo studies.

### Delivery of therapeutic miRs

While chemical modification can improve the stability and reduce off-target effects of miRs, effectively delivering the miRs to the target site is still a major challenge in translating miRs to therapeutics. The intrinsic properties of miRs including hydrophilic nature, negative charge and high molecular weight, reduce the permeability through biological membranes. The primary role of a delivery system is to facilitate the cellular uptake of exogenous miRs to their target sites [206] along with protection from early nuclease degradation, which may affect the target specificity and function of the RNA molecules. Since miRs poses double-stranded (21–23 nucleotides) structure and produces its effects through RISC, the integrity, low cytotoxicity and target specificity is a vital factor to achieve the maximum outcome with a minimum off-target mutation. Besides the obvious requirements of low cytotoxicity and high transfection efficiency, an ideal miRNA delivery system should be able to deliver the miRNA at specific tissue or organ in a local and sustained manner. Despite the effort to develop suitable RNA delivery systems for clinical use, a lack of correlation between in vitro and in vivo efficacy hinders its advancement to the clinic. It is often reported that a delivery system worked efficiently in vitro but failed in vivo due to toxicity, poor pharmacokinetic profiles, nonspecific uptake or immune responses [207], implying that the success of therapeutic miRs is highly dependent on the availability of a safe and efficient delivery system.

#### Non-viral vector delivery system

Most of the non-viral vectors that have been investigated for RNA delivery are also used to deliver other types of nucleic acids including plasmid DNA and antisense oligonucleotides. Lipid and polymer-based delivery systems are the two main categories of synthetic RNA delivery including synthetic-miRs. Apart from its safety profile and low production cost, non-viral vectors can be easily modified by incorporating target ligand to improve delivery efficiency and extend circulation time with the attachment of polyethene glycol (PEG) polymer chains [206]. We will next describe the two commonly used non-viral vector delivery systems (summarized in Table 2).

#### Polymer-based delivery systems

Polymer-based gene delivery system established a great potential for the construction of the ideal gene delivery system. These delivery systems combine their ability to incorporate versatile functional qualities to overcome most of the obstacles encountered in gene delivery. Mainly polymers with redox-sensitive (bio-reducible nature) have gained great attention because of its pivotal role in sustaining cellular homeostasis process [208]. By addition of a disulfide bonds into the basic structure, the polymer holds the nucleic acid load in the extracellular space and selectively release it within the cytosol [208]. Additionally, the introduction of disulfide bonds within these polymers promotes their biodegradability and limits their cytotoxicity. The rational design of polymeric delivery system must consist of condensation and release of nucleic acids in targeted cells or in vivo. Cationic polymers possess the abilities to form compact polymer complex (polyplexes) with nucleic acids and has efficient cell internalization and endosomal escape property [208]. These polymers can form polyplexes through electrostatic interactions with the negatively charged exogenous RNA to facilitate cellular uptake through endocytosis. These are especially designed to protect the nucleic acid when injected into a cell as part of gene therapy [209]. This system retains a net positive charge (ζ-potential), promoting interactions with negatively charged polysaccharides on the cell surface. In addition, polymers that exhibit high proton buffering capacity can promote endosomal escape, thereby avoiding endosomal–lysosomal RNA degradation [210]. Among several cationic polymers, polyethyleneimine (PEI) is one of the widely used polymer for gene delivery. The polyplexes escape from the endosome via proton sponge effect where PEI causes influx of protons and water and endosome swelling and eventually disruption to release the polyplexes to cytoplasm [211]. Synthetic PEI, which has an extensive pH buffering capacity, is the most widely investigated polymer for miR delivery because of its high transfection efficiency [207]. Apart from PEI, dendrimers, which are highly branched synthetic polymers with well-defined molecular architecture, are also frequently studied for nucleic acid delivery [212, 213]. However, because of their high charge density, cationic PEI and dendrimers are often associated with high toxicity thereby limiting their clinical use [214]. Therefore, modified versions of PEI or dendrimers are being developed to address the toxicity issue and to further improve their delivery efficiency [215]. Though PEI mediated miR delivery is very limited, recently PEI has been used to deliver miR-124a (neuro-specific miR) to Neuro2a cells efficiently improved neurogenesis [216]. This delivery provides an efficient intracellular uptake after transfection.

To overcome the toxicity of the polymer-based delivery system, biodegradable poly(lactide-*co*-glycolide) (PLGA) nano-spheres may be helpful, in particular for efficiently delivering cytosolic miRs, which encapsulate miRs by physical entrapment [217–219]. PLGA is an FDA-approved co-polyester that has been widely used in the clinic for drug delivery [220]. Cheng and Saltzman demonstrated that PLGA nanoparticles modified with peptide nucleic acids (PNAs) and phosphorodiamidate morpholino oligomers (PMOs) could load miR inhibitor with high efficiency [221]. In vitro delivery of anti-miR-155 with PMO and PNA modification demonstrated significant knockdown of miR-115 [221] whereas, in vivo study further demonstrated that PLGA nanoparticles encapsulating anti-miR-155 PNA could accumulate in the pre-B cell tumours through enhanced permeability and retention effect [222]. On other hand, Liu et al. demonstrated that anti-miR-204 encapsulated with PLGA nanoparticles promotes osteogenesis in type 2 diabetes [223]. To date only limited reports available for using nanoparticle-mediated delivery of miRs to treat cardiovascular disease. Cetyltriethylammonium bromide (CTAB)-modified positively-charged poly(ethylene oxide)–poly(epsiloncaprolactone) (PEO–PCL) nanoparticles were used to deliver miR-210 into mesenchymal stem cells resulting in a better survival of the cells eventually leading to a better recovery of ischemic rat heart post-transplantation of these cells [224]. However, due to the availability of a variety of nano-carriers capable to effectively transfer miRs, it is not surprising if nanoparticle-mediated delivery of miR-mimics and anti-miRs will be extensively explored in animal models of cardiovascular disease in the near future.

#### Lipid-based delivery systems

Lipid-based miR delivery systems are the most commonly used non-viral vectors in vitro [172]. Like cationic polymers, cationic lipids can form lipo-plexes with extrinsic-RNA through electrostatic interactions. Lipids used for miR-mimics or anti-miRs delivery are composed of a cationic head and a hydrophobic chain. The choice of the head group and the hydrophobic chain may dramatically affect the transfection efficiency and toxicity level of the lipo-plexes. 1,2-Dioleoyloxy-3-trimethylammonium propane (DOTAP) and 1,2-di-*O*-octadecenyl-3-trimethylammonium propane (DOTMA), are frequently used in combination with neutral lipids like dioleoylphosphatidylethanolamine (DOPE) and cholesterol to enhance the transfection efficiency [225]. Incorporation of PEG is a strategy to reduce immunogenicity and maintaining of stability in circulation but glycation of PEG may lead to the reduction of cellular uptake which can be overcome by addition of d-α-tocopheryl (d-α-tocopheryl-PEG succinate) into the delivery system [226, 227]. Currently, there are several commercially available lipid-based delivery systems and most investigations validating miR targets in vitro have used such systems. Wang et al. demonstrated, suppression of miR-320 in MMVEC which has been delivered by cationic lipid base delivery system [82]. On other hand, miRs as a biomarker of heart failure and acute myocardial infarction were validated by transfecting cardiomyoblast cells with three anti-miRs (miR-192, miR-194 and miR-34a) by using cationic lipid base delivery system [228]. Similarly, downregulation of Pim-1 by miR-208a upregulation was validated by anti-miR application through lipid-based delivery system [78].

In comparison to viral vectors, lipid-based delivery systems possess a low range of genetic perturbation, which provides certain benefits to the therapeutic application. However, safe and efficacious delivery in vivo has yet to be achieved due to toxicity, nonspecific uptake, unwanted inflammatory and immune responses [207].

#### Viral vector delivery system

This delivery system works by introduction of viral genome consisting of gene of interest into the cell. This leads to an early phase of gene expression characterized by the appearance of viral regulatory products, followed by a late phase, when structural genes are expressed and assembly of new viral particles occurs. In gene therapy, the viral particles encapsulate with a modified genome, carrying a therapeutic gene cassette in place of the viral genome. This can be achieved by modifying some specific coding regions (may be terminal repeat sequence) from the viral genome, but leaving intact those sequences that are required for packaging the vector genome into the viral capsid. Ideal virus vectors for most gene-delivery system couples the infection pathway but avoid expression of viral genes that leads to replication and toxicity [229]. Viral vectors can successfully transduce the majority of cell types [230, 231]. Viruses that are commonly employed for this purpose include adenovirus, adeno-associated viruses (AAVs), retro-virus and lentiviruses. However, the major drawback with respect to the viral delivery of miR is its relative short half-life due to the susceptibility to degradation by RNase enzyme. Further, safety issues such as potential immunogenicity and insertional mutagenesis remain the main barriers for clinical translation of viral vectors [230, 232]. In addition, low packaging capacity and high production cost have also limited their clinical applications [233, 234].

Adenoviruses are double-stranded DNA viruses, carries approximately 30-kb genome. Recombinant adenoviruses were first used to deliver the gene into the heart in the early 90s [235]. Latter, this delivery system was successfully tested for the cardiac gene in rodents and large animals. Most commonly, adenoviral vectors are used for delivering therapeutic genes into the myocardium with intracoronary infusion, percutaneous or open-chest intra-myocardial injection [236] In fact, they are highly efficient in transducing cardiomyocytes [237, 238], but the transgene expression is temporary and lasts for few weeks [239]. Adenoviral vectors were successfully used into deliver miRs or anti-miRs into rodent models of cardiovascular disease. As for example, downregulation of miR-98 is co-related to myocardial hypertrophy. In contrast, adenovirus-mediated overexpression of miR-98 in cardiomyocytes reduced cell size both at baseline and in response to angiotensin II suggesting a protective role of this miR against angiotensin II-induced cardiac hypertrophy [240]. Similarly, in a mouse model, miR-503-mimic delivery with adenovirus vector improved diabetes mellitus-induced impairment of post-ischemic angiogenesis and blood flow [241]. On other hand, Lu et al. demonstrated that normalization of the miR-328 level with adenovirus-mediated anti-miR reversed atrial fibrillation in wild-type C57BL/6 mice [242]. Adenoviral constructs were also used to investigate a pathogenic role of miR-133 and miR-21 in cardiac hypertrophy [144, 243, 244]. Though adenovirus has been used widely for pre-clinical as well as in clinical studies, due to some sever pitfalls its use has been restricted. In phase I trial of adenoviral gene delivery to treat the malignant intracranial tumour, more serious adverse effects have been reported, such as a severe headache, relapsing seizures, and transient or persistent change of mental status [245].

Adeno-associated virus (AAV) is a non-enveloped virus from the “Parvoviridae” family (a family of small, rugged, genetically-compact DNA viruses) with single-strand DNA genomes of 4.7 kb. To date, 12 primate serotypes have been identified [246] and they are the smallest known viral vectors ideal for the delivery of miR because of the smaller size of miR compared to plasmid-DNA (pDNA). AAVs can transduce both dividing and non-dividing cells and may incorporate its genome into that of the host cell. These features make AAV an attractive candidate for creating viral vectors for gene therapy [247]. Since recombinant AAVs are able to target several tissues, it is crucial to improving tissue specificity, which can be achieved by incorporation into a recombinant AAV of target sequences for tissue-specific miRs [248]. Geisler et al. demonstrated the cardiac-specific miR delivery by using AAV serotype 9 which enable an efficient transduction of the heart upon intravenous injection in adult mice [249]. These data indicate that miR-regulated targeting is a powerful new tool to further improve cardiacspecificity of AAV9 vectors. Karakikes et al. demonstrated AAV9 mediated miR-1 restoration therapeutic strategy to reverse cardiac hypertrophy [250]. On other hand, the number of mitotic cardiomyocytes, positive for H3S10ph, was significantly increased in the hearts of the animals injected with AAV9-miR-590 or AAV9-miR-199a [251]. Moreover, one single intra-cardiac injection of AAV9-miR-590 or AAV9-miR-199a was able to repair the myocardium and restore the cardiac function by improving left ventricular ejection fraction (LVEF) and reduced scar size after 60 days in mice [251]. Furthermore, Chen et al. demonstrated that AAV9 mediated miR-30c delivery into the db/db mice heart markedly attenuate the progression of DCM by inhibition of pathological autophagy [104].

Retroviruses are enveloped viruses that carry two copies of the single-strand RNA genome. Upon cell entry, RNA is copied by the reverse transcriptase enzyme into double-strand DNA. It stably integrates into one of the host chromosomes, assuring long-term expression of inserted therapeutic genes. Viral protein synthesis is not required for retroviral entry and genome integration, therefore all viral genes can be replaced with foreign sequences [252, 253]. To study the importance of miR gene cluster miR-106b-25 (miR-106b, miR-93, and miR-25) in neurogenesis, researchers successfully delivered a retrovirus vector encoding the miR cluster to neural stem/progenitor cells (NSPCs) to improve the lifespan through activation of insulin/IGF signalling in adult stem cells [254]. Retroviruses contain single-stranded, positive-sense RNAs that utilize a virally encoded reverse transcriptase to generate double-stranded DNA. In order to enable viral DNA integration into the host cell genome, the host cell nuclear membrane must be broken down, as occurs during cell division. Retroviruses are therefore limited to transduce dividing cells [255] and cannot efficiently transduce cardiomyocytes. Moreover, due to its non-specificity for the myocardium, this system has not used to deliver miR into the heart.

Lentivirus forms a subgroup of retrovirus that can integrate the exogenous gene into the host genome [256] which enables a stable and a long-term miR silencing effect. Unlike retroviruses, lentiviruses integration within introns of active transcriptional units limits their potential to cause insertional oncogenesis [256, 257]. Due to this property lentiviral vectors are frequently used in regenerative medicine, however, these vectors are capable of transducing mitotically quiescent cells, allowing for efficient transduction of cardiomyocytes. Hence they are frequently used for miR delivery in several cell types including cardiomyocytes.

Yand et al. demonstrated stable downregulation of miR-322 in cardiomyocytes by lentiviral transduction, which prevented hypoxia-induced cardiomyocyte apoptosis [258]. Similarly, Wang et al. showed lentivirus-mediated miR-137 down-regulation reduced apoptosis in cardiomyocytes [259]. Lentiviral-miR-208b transduction was demonstrated to reduce cardiac damage in a myocardial infarction rat model and to inhibit post-infarction myocardial fibrosis, which is possibly achieved via regulation of the important transcription factor, GATA4 [260]. Intra-myocardial injection with lentiviral vector encoding anti-miR-320 promotes downregulation of miR-320 which suppress ischemia–reperfusion-induced apoptosis [81]. Similarly, suppression of miR-34a by lentivirus encoded anti-miR-34a facilitate to improve cardiac contractility after acute myocardial infarction [106].

Moreover, lentiviral-mediated expression of miR-503 in endothelial cells inhibits cell proliferation and migration which makes miR-503 as a possible therapeutic target in diabetic patients with critical limb ischemia [241]. Cordes et al. reported a crucial role of miR-143 and miR-145 in preventing neointima formation caused by proliferation and migration of VSMCs in the rat by lentiviral-mediated transduction [261]. Most of the clinical trials conducted with lentiviral constructs were focused on genetic syndromes. There are no clinical trials evaluating lentiviral constructs in cardiovascular disease. However, due to the great delivery potential, lentivirus-mediated miR delivery has been used widely for the treatment of cardiovascular pathology in pre-clinical level, but the clinical use of lentiviral has been restricted due to the risk of insertional mutagenesis [262, 263].

Besides the common viral vectors, the baculo-viral vector is being increasingly investigated among various viral gene delivery systems. Unlike retroviruses, baculo-viruses neither replicate inside the transduced mammalian cells nor integrate into host chromosomes. Importantly, baculo-viruses are non-pathogenic to humans and baculo-viral DNAs degrade in the mammalian cells over time [132]. It is also demonstrated to transduce a wide variety of stem cells at high efficiency [131]. Although rarely reported as miR carriers for cardiac delivery, due to its high transduction efficacy and low toxicity, baculo-virus are becoming a promising vector for miR delivery in regenerative medicine [133, 134].

However, besides all the advantages, the safety issue is the massive obstacle for viral vector delivery system. Therefore, despite inferior transfection efficiency, non-viral vectors have become attractive alternatives in delivering exogenous miRs due to their better safety profile and lower production cost.

### Assessment of miR modulation and off-target effects

The effect of miR modulation by exogenous miR oligonucleotides can be assessed mostly by hybridization-based assays. First of all, the easy and convenient method appears to be the detection of the fate of target miR. High affinity oligonucleotides, such as LNA/DNA, LNA/2′-OMe and 2′-F/MOE modified anti-miRs, respectively, appropriate the targeted miR in a heteroduplex [186, 190, 203, 264–266], whereas lower affinity oligonucleotides, such as 2′-*O*-Me and 2′-MOE modified anti-miRs and cholesterol-conjugated 2′-*O*-Me antago-miRs, promote miR degradation [203, 266]. Peng et al. demonstrated that stable exogenous anti-miR and endogenous miR hetero-duplexes can be detected using Northern blotting as slower-migrating bands [219]. However, this method is inconvenient due to poor recovery and detection rate of the heteroduplexes [266]. On other hand, the presence of excess exogenous-miR in the RNA sample regardless of any action may interfere with the detection step in real-time qPCR which is misleading for the interpretation of results. Moreover, exogenous-miRs released during RNA extraction facilitates hybridization between the exogenous-miR and endogenous-miR. In order to avoid these drawbacks especially for Northern blot analysis, it has been recommended to use (a) stringent denaturing conditions during electrophoresis [267], (b) increase the hybridization temperature [202], (c) use LNA detection probes [264] or (d) include a competitor probe with an identical sequence as the miR prior to electrophoresis to release the miR from the exogenous and endogenous miR duplex [203, 266]. To overcome those pitfalls, it is always recommended to perform functional assessment after exogenous-miRs application.

The functional readout of miRs modulation has also been assayed by reporter assay of the direct targets. It can be achieved by real time-qPCR, western blot analysis and also through genome-wide transcription assays or by proteomic analysis. This miR-reporter assessment method is based on luciferase or green fluorescence protein (GFP) detection, which has been attached to the 3′-untranslated region (UTR) of a reporter gene. This method has been abundantly used to validate the potency of chemical modification and also the binding ability of exogenous-miRs [190, 197, 198, 264]. On other hand, target mRNA and protein expression show a unique correlation after miR modulation [268]. Thus, an alternative or supplemental approach to employing miR reporter assays is to use the levels of direct target mRNAs and their encoded proteins as functional readouts of miR modulation. However, recent studies demonstrated that changes in mRNA expression levels are only 33–35%, after miR modulation [269, 270] whereas average changes in protein expressions are less than 2-fold following miR modulation [268, 271].

A single miR can regulate multiple targets. Recent advancement in high throughput technique provides the opportunity to analyze all the predicted targets at a time. This technique plays an effective role where single target analysis is unable to provide significant information. Expression microarrays have been widely used in transcriptional profiling experiments and have also been employed in several studies to assess genome-wide transcriptional changes after modulation of miR activity in cultured cells and in vivo [272–275]. Moreover, the recent development in RNA-sequencing technologies has given a breakthrough for genome-wide expression analyses which provides a precise information on alternative splicing, RNA editing and post-transcriptional modification [276]. Additionally, an alternative and impartial method of analyzing the effect of miR perturbation is to use the “Sylamer algorithm” [277] that uses expression changes measured after, for example, miR silencing to rank genes and subsequently test the occurrence of all possible sequence motifs of a given length relative to the sorted gene list.

The use of exogenous-miR for functional miR study or as a therapeutic tool, possess the risk of a mismatch. Thus, understanding the effects of unsolicited interactions between the exogenous-miRs and endogenous nucleic acids is of key importance to minimize off-target effects. Researchers have a dilemma between the length of nucleotides with perfect matching of complimentary sequences. Preferably, longer oligonucleotides are more specific towards complimentary sequences. However, this statement is true only when the hybridization occurred in a controlled condition [202]. In in vivo experiments, the interaction between endo and exogenous-miRs take place in a physiological condition where the chances of imperfect base pairing are very high. To avoid this situation, chemical modifications like LNA-induced-exogenous miRs have been shown to improve mismatch in an efficient way [278]. Seed-targeting anti-miRs predicted to have several complementary sites. Hence, by using Watson–Crick base pairing rules it is possible to identify the number of off-target candidates. MiR-mimics mediated off-target effect was reported by Khan et al. [279], where exogenous miR mediated RISC repel endogenous miR regulation. Similarly, anti-miR administration in cells also demonstrated global saturation effects on endogenous miR targets. However, further research is needed to identify the exact molecular mechanisms leading to the observed effects and to fully understand the consequences of endogenous miR function.

## Conclusion

In just over two decades since their first discovery in humans, miRs have already provided important insight into the biology of several cardiovascular diseases. Their emergent potential as biomarkers in clinical diagnostics as well as modulators for the treatment of a variety of diseases is truly exciting. The potential for these molecules to be used as therapeutic targets for diseases such as DCM is a widely promising research focus, and remains an open question to be answered. From a research perspective, further studies are required to elucidate the exact methods by which miRs are able to modulate translation and initiate mRNA degradation. Similarly, the exact sources, location and role of miRs need to be better defined. Large animal data in cardiovascular field will probably appear as this is worldwide currently under investigation in several laboratories. This will not only result in more insight into their therapeutic potential in larger species but also lead to important information about pharmacokinetics of such exogenous miRs as drugs and safety data. MiR-based therapies are proceeding at a swift rate, with successful completion of phase 1 and phase 2 clinical trials of Santaris Pharma’s LNA-modified anti-miR, targeting miR-122 for the treatment of hepatitis C virus infection [280]. In future this may pave the way for large-scale mechanism orientated miR based therapeutic trials in cardiovascular medicine. Additionally, technological advances are required to enable rapid, reliable and reproducible results for the absolute quantification of miRs to facilitate transition into clinical practice. Finally, while there remain a number of challenges to overcome, clinicians should be aware of the potential arrival of miR-based therapeutics into the domain of clinical medicine.
